# Supplementary material for: Osteolytic Bone Loss and Skeletal Deformities in a Mouse Model for Early-Onset Paget’s Disease of Bone with PFN1 Mutation Are Treatable by Alendronate
Source: Pharmaceuticals (Basel). 2023 Oct 2;16(10):1395. doi: 10.3390/ph16101395 (PMC10610320; doi:10.3390/ph16101395)
Supplement: Supplementary file 1 [file pharmaceuticals-16-01395-s001.zip › pharmaceuticals-2612786-supplementary.pdf]

Fig. S1

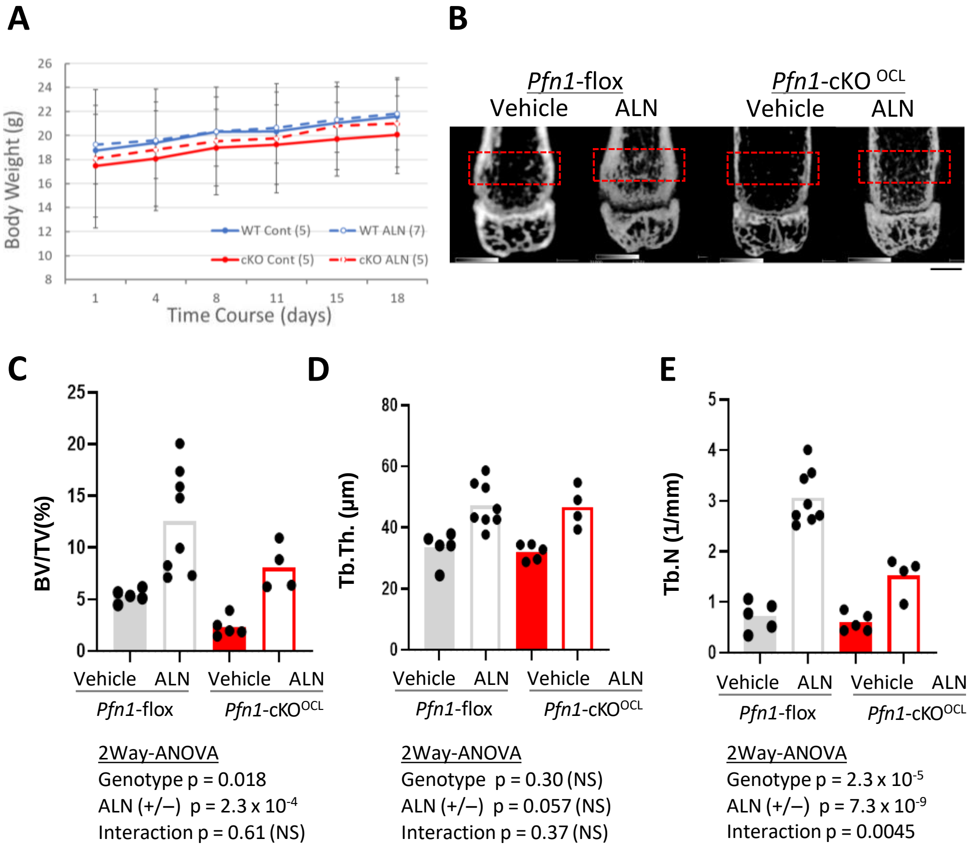

Figure S1. Bone mass analysis of *Pfn1*-cKO^{OCL} mouse femurs (males) **A**. Chronological changes in body weight gain were plotted on the graph. **B**. Representative µCT images of distal femurs in four experimental groups. Scale bar: 1 mm **C-E**. Trabecular bone mass at distal femur metaphysis was analyzed using 3D µCT with standard parameters, i.e., BV/TV (C), Tb.Th. (D), and Tb.N. (E) respectively.

Fig. S2

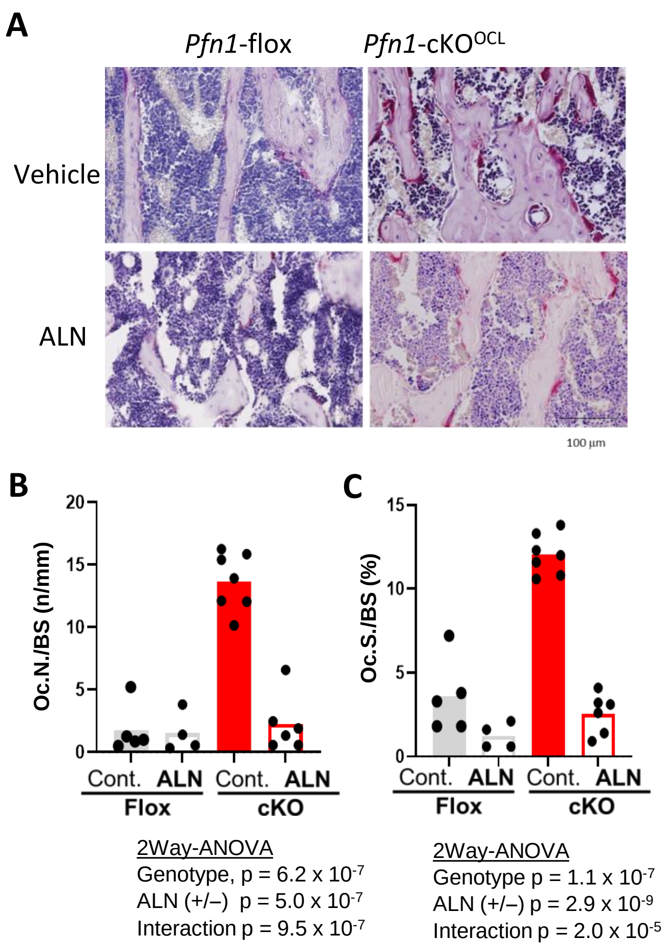

Figure S2. Bone resorptive parameters for proximal tibias by TRAP staining. A. Representative microscopic images for proximal tibia by frontal section. Multi-nucleated red cells with more than 2 nuclei were regarded as TRAP-positive osteoclasts. Scale bar: 100 µm **B and C**. Bone resorptive parameters were plotted on the graphs for OcN./BS (B), and OcS./BS (**C**), respectively.

Fig. S3

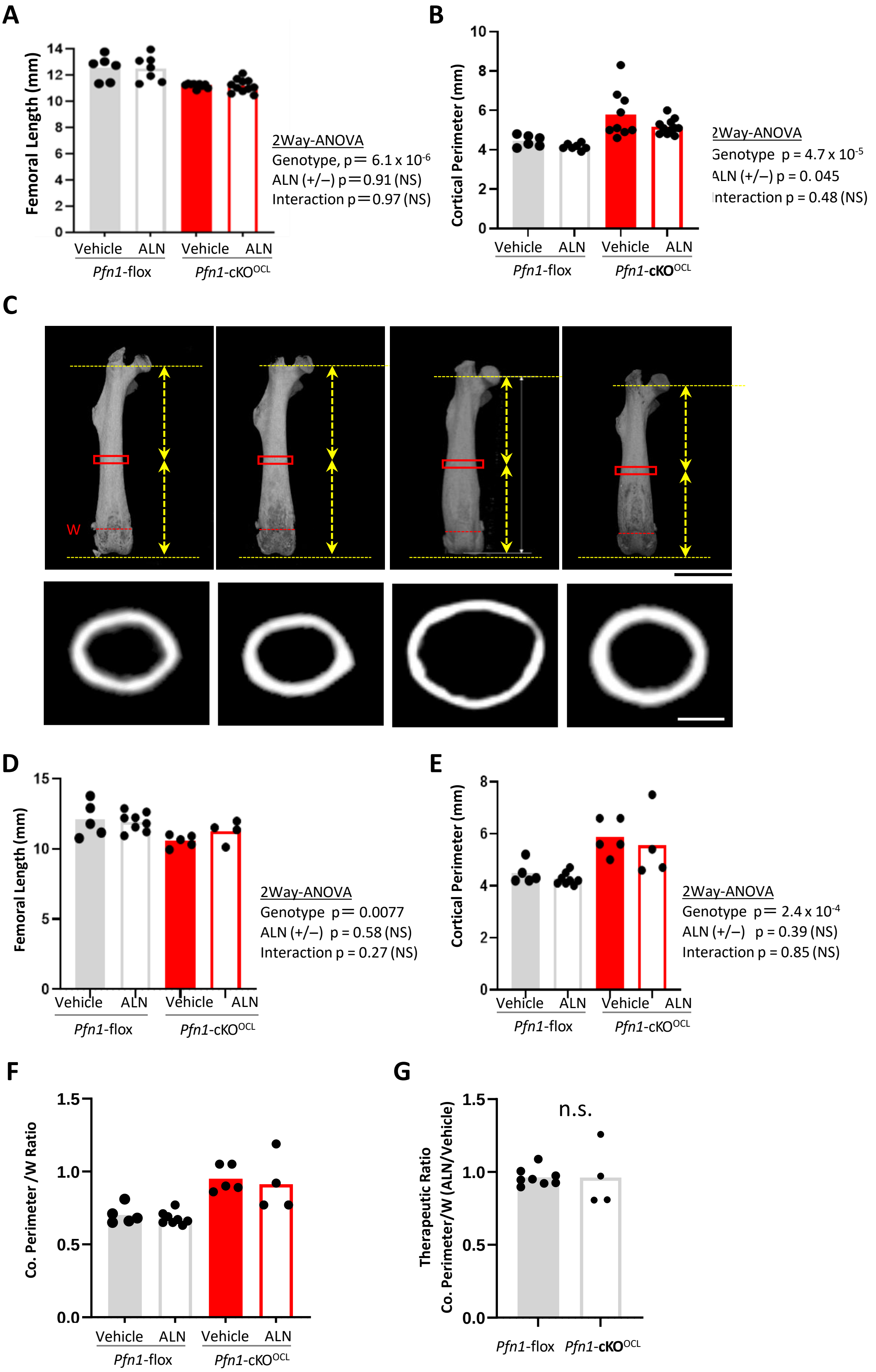

A
Femoral Length (mm)
Vehicle ALN Vehicle ALN
Pfn1-flox Pfn1-cKO^OCL
2Way-ANOVA
Genotype, p= 6.1 x 10^-6
ALN (+/–) p=0.91 (NS)
Interaction p=0.97 (NS)
B
Cortical Perimeter (mm)
Vehicle ALN Vehicle ALN
Pfn1-flox Pfn1-cKO^OCL
2Way-ANOVA
Genotype p = 4.7 x 10^-5
ALN (+/–) p = 0. 045
Interaction p = 0.48 (NS)
C
W
D
Femoral Length (mm)
Vehicle ALN Vehicle ALN
Pfn1-flox Pfn1-cKO^OCL
2Way-ANOVA
Genotype p= 0.0077
ALN (+/–) p = 0.58 (NS)
Interaction p = 0.27 (NS)
E
Cortical Perimeter (mm)
Vehicle ALN Vehicle ALN
Pfn1-flox Pfn1-cKO^OCL
2Way-ANOVA
Genotype p= 2.4 x 10^-4
ALN (+/–) p = 0.39 (NS)
Interaction p = 0.85 (NS)
F
Co. Perimeter /W Ratio
Vehicle ALN Vehicle ALN
Pfn1-flox Pfn1-cKO^OCL
G
Therapeutic Ratio
Co. Perimeter/W (ALN/Vehicle)
n.s.
Pfn1-flox Pfn1-cKO^OCL

Figure S3. Analysis of the size and cortical parameters of the femurs in WT and *Pfn1*-cKO^{OCL} mice (females and males) **A**. The length of the femoral shaft as indicated in Fig. 3A was compared on the graph (females). **B**. The cross-sectional cortical perimeter (Ct.Pm) of the femur at midshaft was plotted on the graph (females). **C**. Representative μCT images of the male femurs in AP projection (upper panels) and in cross-section at midshaft (bottom) that were analyzed for cortical bone parameters in D-F. Red boxes indicate the ROI for cortical bone analysis. Vertical arrows indicate the length of the proximal and distal halves of the femoral shaft. Horizontal arrows indicate the width (W) of the border between the distal metaphysis and the epiphyses at the growth plate level. Scale bar: 0.5 mm **D**. The length of the femoral shaft as indicated in C was compared on the graph (males). **E**. The cross-sectional cortical perimeter of the femurs was plotted on the graph (males). **F**. Cortical Perimeter (Ct.Pm) normalized by epiphyseal width (W) were plotted on the graph. **G**. The therapeutic ratio of the above index by ALN was plotted on the graph to compare it between the WT and *Pfn1*-cKO^{OCL} mice by Student's t-test (**; $p<0.01$).

Fig. S4

A

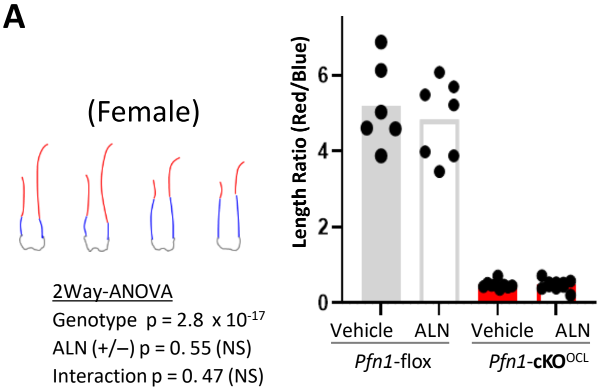

B

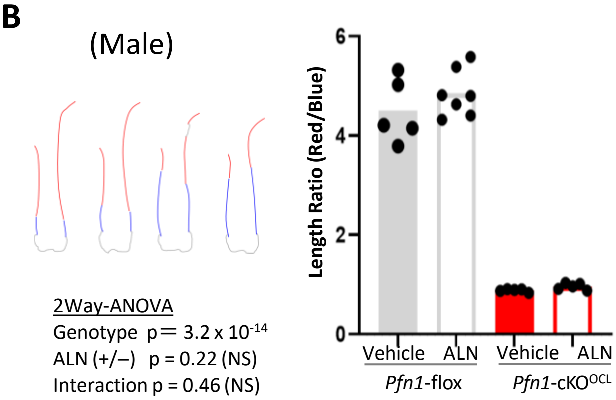

Figure S4. Morphometric analysis of *Pfn1*-cKO^{OCL} mouse femurs at midshaft (males). **A**. The length ratio for the concave versus convex curves around the midshaft was calculated in each group. Representative traces for the contour curves are shown at the left (females) **B**. The length ratio for the concave versus convex curves around the midshaft was calculated in each group. Representative traces for the contour curves are shown at the left (males).

Fig. S5

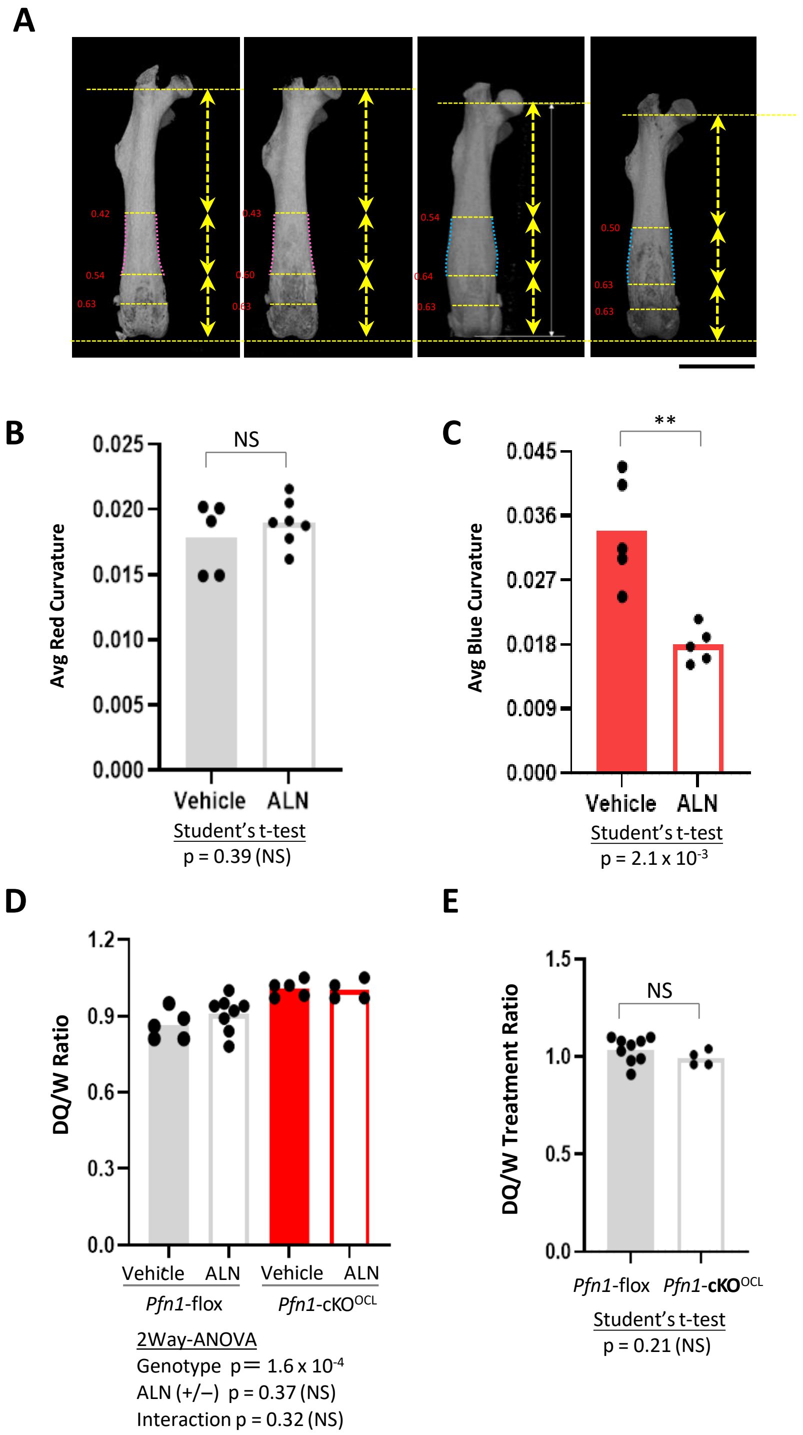

Figure S5. Geometric analysis of *Pfn1*-cKO^{OCL} femurs from midshaft to the distal metaphysis (males) **A**. The outline of the AP projection μCT images represented in Fig. S4 was traced with pink and light blue curves from the midshaft to the distal quartile or distal growth plate, indicating concave and convex features, respectively in diaphysis to metaphysis (upper panels). Vertical arrows indicate the length of the proximal half, third and fourth quartile. Horizontal arrows indicate the mediolateral width of the distal quartile (DQ), and the distal growth plates (W). Scale bar: 1 mm **B**. The average curvature in concave contour (pink curves) was compared between the control versus ALN-treated WT mice. **C**. The average curvature in convex contour (light blue curves) was compared between the control versus ALN-treated *Pfn1*-cKO^{OCL} mice. **D**. The distal quartile expansion parameter was calculated as the DQ/W ratio and analyzed by plotting and comparing among the groups. **E**. Relative effect of ALN treatment in the DQ/W in panel D was statistically analyzed by Student's t-test. (**; p<0.01)

Fig. S6

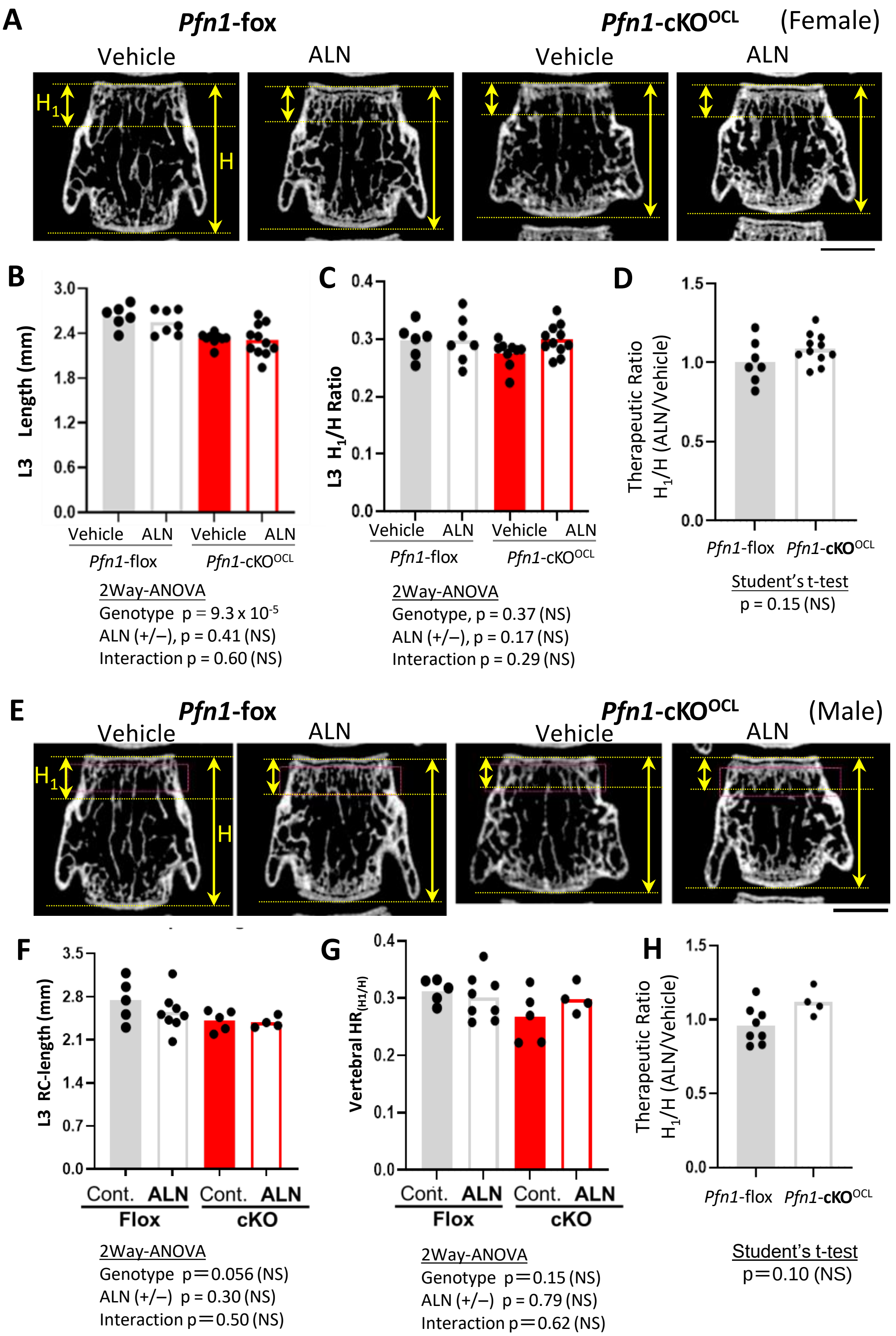

Figure S6. Bone shape analysis of *Pfn1*-cKO^{OCL} L3 vertebrae **A**. Representative 2D μCT images of frontal sections of the L3 vertebral body were shown to explain the anatomical sites of the length analysis (female). Scale bar: 1 mm **B**. The rostrocaudal length of the L3 vertebral body (L3 length =H) was compared among the groups by 2-way ANOVA. **C**. The relative length of the distal portion of the L3 vertebral body (H1) was normalized by H and compared among the groups similarly. **D**. The ratio of therapeutic effect by ALN for the H1/H ratio was compared by Student's t-test. NS; not significant. **E**. Representative 2D μCT images of frontal sections of the L3 vertebral body were shown to explain the anatomical sites of the length analysis (male). Scale bar: 1 mm **F**. The rostrocaudal length of the L3 vertebral body (L3 length =H) was compared among the groups. **G**. The relative length of the distal portion of the L3 vertebral body (H1) was normalized by H and compared among the groups. **H**. The ratio of therapeutic effect by ALN for the H1/H ratio was compared by Student's t-test. NS; not significant.

Fig. S7

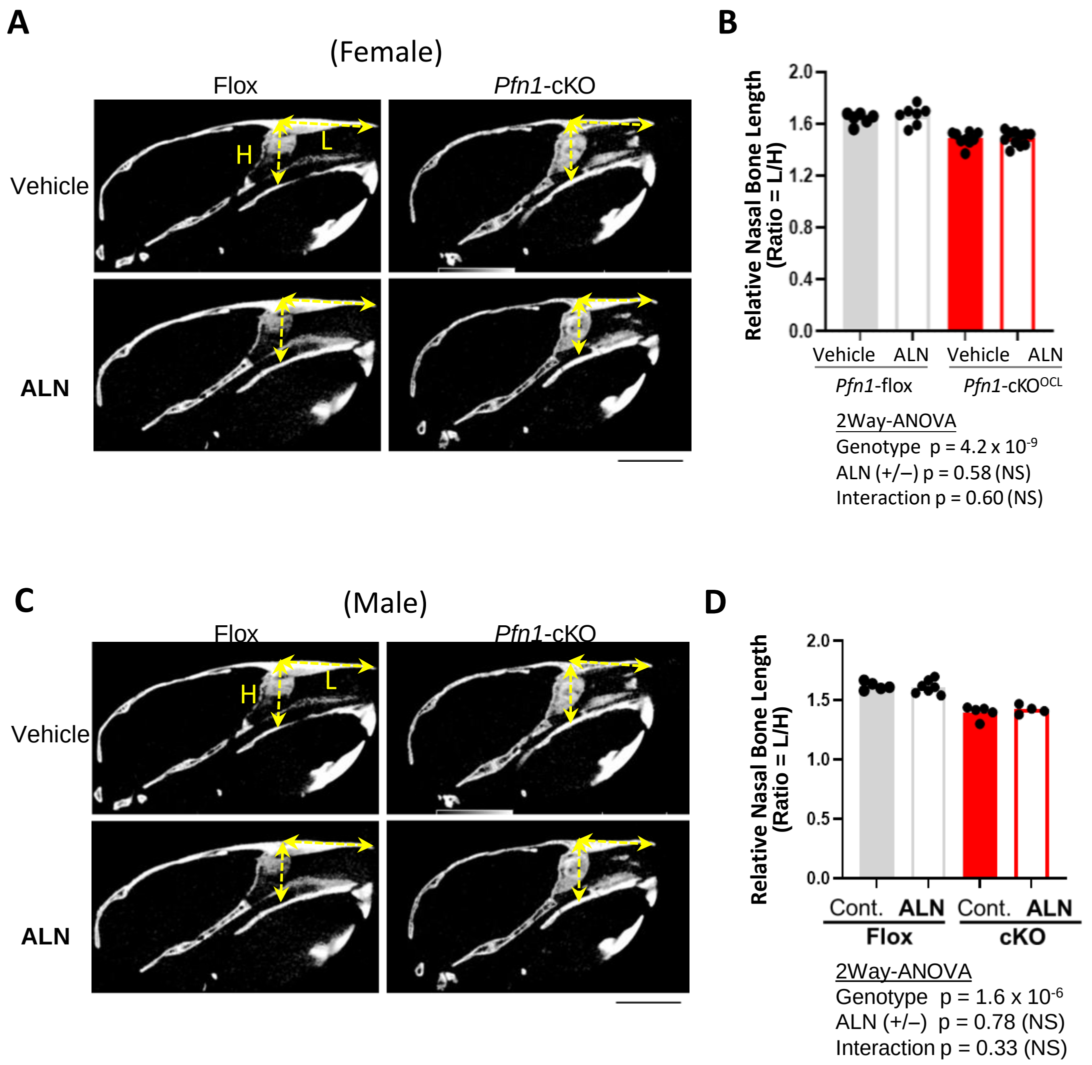

Figure S7. Bone shape analysis of *Pfn1*-cKO^{OCL} Skull **A**. Representative 2D µCT images of sagittal sections of the skull were shown to explain the anatomical sites of the length analysis (female). Scale bar: 1 mm **B**. The relative length of nasal bone was compared between the groups. **C**. Representative 2D µCT images of sagittal sections of the skull were shown to explain the anatomical sites of the length analysis (male). Scale bar: 1 mm **D**. The relative length of nasal bone was compared between the groups.

Table S1

Summary of the trabecular bone mass parameters at distal femur metaphysis (Male).

	Pfn1-flox		*Pfn1*-cKO^{OCL}		p-values/ 2-Way ANOVA		
	**Vehicle (6)**	**ALN (7)**	**Vehicle (9)**	**ALN (11)**	**Genotype**	**ALN (+/-)**	**Interaction**
**BV/TV (%)**	3.87 ± 1.28	14.0 ± 5.28	1.43 ± 0.85	8.08 ± 2.23	0.013	2.0 x 10^{-5}	0.27
**Tb.N (/mm)**	0.83 ± 0.11	3.06 ± 0.53	0.42 ± 0.29	1.52 ± 0.38	2.3 x 10^{-5}	7.3 x 10^{-9}	0.005
**Tb.Th (μm)**	43.1 ± 10.9	47.3 ± 7.22	36.4 ± 3.81	46.7 ± 6.62	0.3	0.057	0.37
**Tb.Sp (μm)**	859.5 ± 470.1	288.9 ± 129.4	3699 ± 2732	520.3 ± 43.0	0.018	0.009	0.036
**Tb.Spac (μm)**	822.7 ± 580.0	329.8 ± 139.7	3736 ± 2735	549.5 ± 76.7	0.017	0.011	0.033

Table S2

Summary of the trabecular bone mass parameters at the third vertebral body (Male).

	Pfn1-flox		*Pfn1*-cKO^{OCL}		p-values/ 2-Way ANOVA		
	**Vehicle (6)**	**ALN (7)**	**Vehicle (9)**	**ALN (11)**	**Genotype**	**ALN (+/-)**	**Interaction**
**BV/TV (%)**	7.86 ± 2.36	13.2 ± 5.91	1.46 ± 0.97	7.99 ± 0.69	3.3 x 10^{-3}	2.8 x 10^{-3}	0.74 (NS)
**Tb.N (/mm)**	3.87 ± 1.50	4.74 ± 1.42	0.81 ± 0.45	3.33 ± 0.32	4.3 x 10^{-4}	6.3 x 10^{-3}	0.13 (NS)
**Tb.Th (μm)**	22.8 ± 0.97	26.7 ± 4.57	17.9 ± 1.41	23.9 ± 0.95	0.01	1.5 x 10^{-5}	0.44 (NS)
**Tb.Sp (μm)**	291.4 ± 103.8	205.9 ± 91.7	1254 ± 506.9	277.6 ± 28.8	2.4 x 10^{-4}	5.2 x 10^{-4}	7.7 x 10^{-4}
**Tb.Spac (μm)**	314.2 ± 104.3	232.7 ± 88.2	1276 ± 504.6	301.5 ± 29.2	2.4 x 10^{-4}	5.2 x 10^{-4}	7.2 x 10^{-4}
